# Selective and Accurate Detection of Nitrate in Aquaculture Water with Surface-Enhanced Raman Scattering (SERS) Using Gold Nanoparticles Decorated with β-Cyclodextrins

**DOI:** 10.3390/s24041093

**Published:** 2024-02-07

**Authors:** Zhen Li, Yang Hu, Liu Wang, Houfang Liu, Tianling Ren, Cong Wang, Daoliang Li

**Affiliations:** 1 National Innovation Center for Digital Fishery, China Agricultural University, Beijing 100083, China; 2 School of Integrated Circuit, Tsinghua University, Beijing 100084, China; 3 Key Laboratory of Smart Farming Technologies for Aquatic Animal and Livestock, Ministry of Agriculture and Rural Affairs, China Agricultural University, Beijing 100083, China; 4 College of Information and Electrical Engineering, China Agricultural University, Beijing 100083, China; 5 Beijing National Research Center for Information Science and Technology (BNRist), Tsinghua University, Beijing 100084, China

**Keywords:** β-cyclodextrin, gold nanoparticles, SERS, nitrate nitrogen detection, aquaculture water

## Abstract

A surface-enhanced Raman scattering (SERS) method for measuring nitrate nitrogen in aquaculture water was developed using a substrate of β-cyclodextrin-modified gold nanoparticles (SH-β-CD@AuNPs). Addressing the issues of low sensitivity, narrow linear range, and relatively poor selectivity of single metal nanoparticles in the SERS detection of nitrate nitrogen, we combined metal nanoparticles with cyclodextrin supramolecular compounds to prepare a AuNPs substrate enveloped by cyclodextrin, which exhibits ultra-high selectivity and Raman activity. Subsequently, vanadium(III) chloride was used to convert nitrate ions into nitrite ions. The adsorption mechanism between the reaction product benzotriazole (BTAH) of o-phenylenediamine (OPD) and nitrite ions on the SH-β-CD@AuNPs substrate was studied through SERS, achieving the simultaneous detection of nitrate nitrogen and nitrite nitrogen. The experimental results show that BTAH exhibits distinct SERS characteristic peaks at 1168, 1240, 1375, and 1600 cm^−1^, with the lowest detection limits of 3.33 × 10^−2^, 5.84 × 10^−2^, 2.40 × 10^−2^, and 1.05 × 10^−2^ μmol/L, respectively, and a linear range of 0.1–30.0 μmol/L. The proposed method provides an effective tool for the selective and accurate online detection of nitrite and nitrate nitrogen in aquaculture water.

## 1. Introduction

As aquacultures undergo continual development towards intensification and larger-scale operations, the imperative for a sustainable, environmentally friendly, and health-focused development model emerges as the guiding principle for the future of this industry. Aquaculture water quality plays a pivotal role in influencing the healthy growth of aquaculture organisms and remains the primary contributor to harmful pollutant residues in aquatic products. Nitrate nitrogen, a common nitrogen-containing waste in aquaculture water, primarily originates from fish bait and the waste generated by aquaculture organisms. Although the toxicity of nitrate nitrogen is relatively lower than that of nitrite nitrogen and ammonia nitrogen, prolonged exposure to excessive nitrate levels can have adverse effects on aquatic animals, affecting their health and growth rates [1]. Additionally, nitrate nitrogen has the potential to convert into nitrite nitrogen and ammonia nitrogen, with nitrate being reduced to the more reactive and toxic nitrite nitrogen, posing further risks to aquaculture organisms. The existing literature substantiates the idea that the extensive utilization of nitrogen-based fertilizers, including ammonium nitrate, potassium nitrate, and sodium nitrate, is contributing to the decline of amphibians [2]. Therefore, the precise detection of nitrate nitrogen in aquaculture water is a crucial matter that warrants attention.

Traditional methods for detecting nitrate nitrogen include the ion-selective electrode method (ISE), Griess reagent method, gas-phase molecular absorption spectrometry, and ultraviolet spectrophotometry [3,4,5]. The spectrophotometric method based on the Nessler reagent is currently the most commonly used detection method, but it requires large instruments and a complex preprocessing process [6]. Therefore, it is not suitable for the detection of complex water environments. Recently, some electrochemical [7,8] and optical [9,10] detection methods have been increasingly reported for nitrate detection in water. Electrochemical sensors represent the most widely used and mature technology in this domain. ISE is one of the potentiometric methods for detecting nitrate in electrochemical methods [11,12]. Sephra et al. [13] developed a nitrate ion-selective electrode (NO_3_-ISE) based on Ni_2_O_3_/rGO nanocomposites (NNGC) for the detection of nitrate ions. The ISE method for detecting nitrites has the advantages of high selectivity, real-time monitoring, and high sensitivity [14]. However, it comes with the drawbacks of higher costs associated with ion-selective electrode preparation and maintenance, potential interference from other substances, and a limited linear range. Voltage amperometry is a widely applicable and sensitive electrochemical method, particularly suitable for the development of portable sensors in an aquaculture [15,16], such as screen-printed electrodes (SPEs) [17]; however, it exhibits relatively lower specificity and may be influenced by sample complexity and operational intricacies. A current research focus in nitrate detection lies in the nanomaterial-modified electrode method, such as metal nanoparticles, carbon-based nanomaterials, and graphene, which have excellent electrocatalytic performance, contributing to improving the electrochemical signal of nitrates and enhancing detection sensitivity [18,19], offering enhanced selectivity and sensitivity. However, challenges persist in addressing the stability and reproducibility of nanomaterials, necessitating further exploration and development in this area.

Additionally, optical detection methods, such as UV and mass spectrometry, are also widely used in a water quality analysis due to their ultra-high sensitivity and environmental adaptability [9,20]. Fluorescence detection methods have also been reported [9]. However, the current optical methods, specifically the spectrophotometric method, are operationally complex and susceptible to interference from other ions when applied to actual aquaculture water [21]. Raman spectroscopy is also one of the optical detection methods, with advantages such as high sensitivity, high selectivity, real-time detection, label-free operation, and a fast response [22,23]. The outstanding fingerprint recognition ability of surface-enhanced Raman spectroscopy (SERS) has found extensive applications in chemistry, biology, environmental science, and other fields, particularly in the detection and analysis of trace substances [24,25].

In the field of water quality monitoring, SERS is currently employed for the detection of various pollutants in water, such as heavy metal ions [26,27], organic compounds [28,29], and microorganisms [30], providing the rapid monitoring of water pollution. The detection of nitrate nitrogen in water using Raman spectroscopy techniques, especially the application of SERS, has been reported. SERS has proven to be particularly effective for measuring nitrate and nitrite nitrogen in aquaculture water [31,32]. In a previous report, we introduced a SERS detection method for nitrites based on a single metal nanoparticle substrate, achieving favorable detection results [33]. Nanoparticles, with their unique photoelectrochemical properties, have been extensively used in various optical sensors, electrochemical sensors, and biosensors [34,35,36]. In SERS applications, single metal nanoparticle substrates have limitations in stability, relatively limited surface enhancement effects, and low specificity. Modifying the surface of nanomaterials, with advantages such as enhanced stability, controlled surface enhancement effects, and improved specificity, is more suitable for dealing with complex environments and meeting sensitivity requirements [32]. The sensitivity and anti-interference capabilities of single gold (Au) and silver (Ag) nanomaterial substrates for SERS detection of low-concentration nitrate nitrogen are insufficient, and portable Raman spectrometers may not achieve the required accuracy. Currently, SERS nanocomposites substrates generally include metal alloy particles, core–shell structures, composite material nanostructures, molecularly imprinted polymer composites, and two-dimensional material composites [37,38,39,40,41]. Additionally, the combination of supramolecular compounds with metal nanomaterials broadens the application of SERS [42]. Therefore, the development of highly sensitive and selective SERS substrates is crucial for achieving the rapid and accurate detection of low-concentration nitrate nitrogen in aquacultures.

Cyclodextrins, a class of cyclic oligosaccharides, are compounds characterized by a macrocyclic ring of glucose substituents linked by α-1,4 glycosidic bonds, rendering them supramolecular compounds [43,44,45,46]. Cyclodextrins are produced by the enzymatic conversion of starch, while typical cyclodextrins contain many glucose monomers, with β-cyclodextrin, featuring seven glucose subunits, being specifically employed in this study. The unique structural characteristics of cyclodextrins confer strong hydrophobicity to their inner regions, while the outer side exhibits robust hydrophilicity due to a multitude of hydroxyl aggregates [47]. β-cyclodextrin molecules, owing to their structural characteristics, possess the capability to efficiently encapsulate various target substances within their cavities. This property has rendered them widely employed in the realms of both chemistry and biology [48,49,50,51,52,53]. The combination of metal nanoparticles with supramolecular compounds such as cyclodextrins can effectively identify the target substance and has been widely used in molecular recognition and photoelectrochemical sensors [42,54,55,56,57]. Building upon this principle, this study explores cyclodextrin-encapsulated gold nanoparticles to enhance the selectivity and Raman activity of a single metal nanomaterial substrate. The substrates exhibit a high SERS enhancement factor, along with ultra-high sensitivity and selectivity, facilitating the rapid and accurate detection of nitrate nitrogen in aquaculture water.

In this paper, a nanomaterial substrate functionalized with cyclodextrin-encapsulated gold nanoparticles (SH-β-CD@AuNPs) was prepared by reacting o-phenylenediamine (OPD) with nitrite, incorporating mono-(6-mercapto)-β-cyclodextrin molecules and gold nanoparticles. This nanomaterial substrate served as a highly effective tool for the measurement of nitrate nitrogen at low concentrations in aquaculture water. First, nitrate was oxidized to nitrite by vanadium (III) chloride (VCl_3_), followed by the cyclization of nitrite and OPD under acidic conditions to form benzotriazole (BTAH). BTAH, exhibiting high SERS activity on SH-β-CD@AuNPs substrates, enabled the indirect detection of nitrate nitrogen by analyzing the SERS spectra of BTAH. The method investigated in this paper combines cyclodextrins with metal nanoparticles to prepare SH-β-CD@AuNPs nanomaterial substrates and demonstrated remarkable accuracy in the measurement of nitrate nitrogen in aquaculture water. The experimental results additionally demonstrate that the method presented in this paper holds broader applicability in the field of nitrate nitrogen and nitrite nitrogen detection.

## 2. Materials and Methods

### 2.1. Reagents

Chloroauric acid (HAuCl_4_, 99.9%), sodium carbonate (NaCO_3_, 99.0%), sulfamic acid (NH_2_SO_3_H, 99.0%), borax (Na_2_BO10H_2_O, 99.0%), sodium bicarbonate (NaHCO_3_, 99.0%), and sodium hydroxide (NaOH, 99.0%) were purchased from Aladdin Reagent Co., Ltd. (Shanghai, China). O-phenylenediamine (C_6_H_8_N_2_, 98.5%), vanadium(III) chloride (VCl_3_, 99.0%), and β-cyclodextrin (C_42_H_70_O_35_, 99.0%) were obtained from Macklin Biochemical Technology Co., Ltd. (Shanghai, China). Hydrochloric acid (HCl, 36–38%, AR) was purchased for Sinopharm Chemical Reagent Co., Ltd. (Shanghai, China). All other reagents were obtained from Shanghai Chemical Reagent Co. (Shanghai, China), and all solutions were stored in the dark at 4 °C. All other reagents were of analytical pure grade and above, and water for all solutions was prepared using deionized water (=18.20 MΩ cm).

### 2.2. Instruments

Ultra-Visual (UV–Vis) absorption spectroscopy was employed to determine the absorption of the nanoparticles and reaction products (INESA-757, INstruments and Electronics Associates, Shanghai, China). Analysis of AuNPs and modified nanomaterial substrate was performed using transmission electron microscope (TEM, JEM-2100F, JEOL, Tokyo, Japan) (Malvern, Zetasizer nano ZS90, Malvern, UK). A spectrophotometer system (DR 3900, Hach, Loveland, CO, USA) was used to confirm the results. The CNC ultrasonic cleaner, magnetic stirrer, high-speed centrifuge, drying oven were used for the fabrication of nanomaterials. Additionally, the QE-Pro Raman spectrometer analysis system of Ocean Optics was used, the excitation light source was 785 m, equipped with RPB-785-1.5-SS Raman fiber-optic probe and cuvette, and the software used was Oceanview1.6.

### 2.3. Samples

Four kinds of water samples, such as tap water, aquaculture water in fish tank (carp), aquaculture water in land-based factories (grouper), and seawater, were prepared to validate the present method. The tap and the tank water were collected in the laboratory (the tap and the tank water were collected in the laboratory (China Agricultural University National Innovation Center for Digital Fishery, Beijing, China)), and the seawater was gathered from the sea near Mingbo, Yantai, Shandong Provence, China. The aquaculture water in land-based factories was provided by Mingbo Aquatic, Shandong Provence, China. The water samples were filtered with 0.45 μm microporous membranes to eliminate possible interference caused by turbidity due to particles. Real water samples were obtained by adding nitrate standard solutions with concentrations of 0 and 5 μM to aquaculture water, which were used for the evaluation of feasibility.

### 2.4. Solution Preparation

For OPD stock solutions (10^−4^ mol/L), 0.1081 g of OPD was dissolved in 10 mL deionized (DI) water under heating conditions. Sodium nitrate (NaNO_3_) standard stock solutions (10^−2^–10^2^ mol/L) were prepared by dissolving 0.085 g of sodium nitrate solid powder in 10 mL DI water, and store in dark place at 4 °C. The other concentrations of sodium nitrate working solution were sequentially diluted with deionized water. HCl working solutions were prepared by taking 1 mL of HCl (37%) into 10 mL DI water. VCl_3_ stock solutions were prepared by dissolving 0.08 g of VCl_3_ solid and adding 0.8 mL of HCl working solution into 10 mL DI water. Sulfamic acid working solutions (6 × 10^−4^ mol/L) were obtained by dissolving 0.058 g of sulfamic acid in 10 mL DI water. Cyclodextrin working solutions were prepared by dissolving 0.01 g of cyclodextrin in 10 mL DI water. Chloroauric Acid Solutions (1%) were made by dissolving 0.5 g chloroauric acid solid in 50 mL DI water. Dilutions of other concentration solutions were made using deionized water.

### 2.5. Preparation of Modified Nanomaterial Substrate

#### 2.5.1. Fabrication of AuNPs

In this paper, Au nanoparticles (AuNPs) with particle sizes of 55 nm, were prepared with sodium citrate reduction method [58,59], respectively. The steps of synthesis are as follows: a 100 mL solution of 0.01% chloroauric acid was heated to boiling in a round-bottom flask with continuous stirring and a rapid addition of 1.8 mL of 1.0% sodium citrate solution, resulting in a color change from black to burgundy. The solution was heated for an additional 30 min, cooled, and then restored to its original volume with deionized water, yielding 50 nm gold nanoparticles. For larger size AuNPs preparation, the detailed preparation process has been reported in our previous paper [33,60,61]. The AuNPs were put into 50 mL brown glass vials cooled at room temperature and finally transferred to the refrigerator for storage at 4 °C. All glassware should be kept clean during nanomaterial synthesis.

#### 2.5.2. Fabrication of β-Cyclodextrin-Modified AuNPs

The cyclodextrin employed in this paper was mono-(6-mercapto)-β-cyclodextrin. Consequently, the experiment involved the modification of SH-β-CD@AuNPs with cyclodextrin sulfhydryl groups. It is known that the sulfhydryl groups on cyclodextrins readily adhere to the surface of AuNPs, forming S-Au bonds and thus ensuring the stable modification of cyclodextrins onto the AuNPs [62,63]. The method employed in this paper involved the use of β-CD as a reducing and stabilizing agent for the preparation of SH-β-CD@AuNPs.

The process for preparing AuNPs consistently utilized sodium citrate as both the reducing agent and stabilizer. As reported in the literature, mono-(6-mercapto)-β-cyclodextrin, comprising D-D-glucopyranose, exhibits inherent reducing properties. Hence, in the experiment, cyclodextrin also functioned as a reducing and stabilizing agent for the direct synthesis of SH-β-CD@AuNPs. The preparation method closely mirrored that of AuNPs synthesis. Specifically, a solution of 0.1% chloroauric acid was heated and stirred, and a 1 g/L concentration cyclodextrin solution was swiftly introduced with continuous stirring. This mixture was maintained in an 80 °C water bath for 10 min, resulting in a burgundy-colored solution. The final solution was cooled to room temperature, and the prepared nanomaterials were stored in brown glass vials in a refrigerator at 4 °C. It is noteworthy that cyclodextrin-coated gold nanoparticles tend to aggregate when the particle size exceeds 50 nm. The resultant mixed solution exhibited a burgundy-red color, and the final pH of the solution was 7.0.

### 2.6. Experimental Steps

The traditional cadmium column reduction method for nitrate reduction is intricate in its procedures and involves the use of toxic reagents. Conversely, the ultraviolet reduction method is both inefficient and less stable. In this study, an innovative approach was adopted, replacing VCl_3_ for the conventional cadmium column to reduce nitrate to nitrite. Specifically, a 5 mL centrifuge tube was loaded with 200 μL of nitrate solution. Subsequently, 100 μL of VCl_3_ working solution was incrementally added, followed by dilution to 1 mL with deionized water. The resulting mixture was sealed and stored. This composite solution was placed in a desiccator at 50 °C for 35 min to ensure the complete conversion of nitrate to nitrite. Following this, 200 μL of OPD solution was introduced to the composite solution and allowed to react at room temperature for 15 min. Upon completion of the reaction between OPD and nitrite, 0.5 mL SH-β-CD@AuNPs solution was added to the mixture. After shaking for 1 min, the volume was adjusted to 2 mL, and the solution was left at room temperature for an additional 2 min to ensure the thorough adsorption of the generated BTAH and the SH-β-CD@AuNPs substrate. Ultimately, the mixed solution was transferred to a 1 cm quartz cuvette for SERS detection. The experimental dosage ratio for sodium nitrate solution, VCl_3_ solution, OPD solution, and SH-β-CD@AuNPs substrate was maintained at 2:1:2:5.

In the presence of both nitrate and nitrite ions, aminosulfonic acid can be employed to eliminate the interference from nitrite ions. However, the use of aminosulfonic acid introduces some margin of error, influencing the experimental results. Therefore, when conducting experiments under conditions of coexistence of nitrate and nitrite ions, we simultaneously measured two sets of comparative samples during the measurement. Hence, the expression for the detection of ${{NO}_{3}}^{-}$ is given by

(1) $${{NO}_{3}}^{-}={NO}_{Xmeasure}-{{{NO}_{2}}^{-}}_{measure}$$

where ${NO}_{Xmeasure}$ is the detected concentration (μmol/L) when ${{NO}_{2}}^{-}$ and ${{NO}_{3}}^{-}$ in the sample are converted to ${{NO}_{2}}^{-}$, and ${{{NO}_{2}}^{-}}_{measure}$ is the concentration of nitrite ions in the original sample (μmol/L).

Additionally, in this experiment, the excitation wavelength was selected to be 785 nm, the laser power selected to be 300 mW, the exposure time was selected to be 5 s, and the number of detections was selected to be 5 times for the SERS detection of nitrate nitrogen based on SH-β-CD@AuNPs substrate.

## 3. Results and Discussion

### 3.1. Principle of Nitrate Detection Based on SH-β-CD@AuNPs

The Raman signal of nitrate ions is inherently weak, even at high concentrations, making it challenging to observe their Raman characteristic peaks. While SERS signals for high concentrations of nitrate ions may reveal characteristic peaks, practical detection in aquaculture water with low nitrate ion concentrations (1 mg/L) often results in weak SERS signals, leading to a suboptimal detection performance. In this study, VCl_3_ was employed to convert nitrates into nitrites. The principle is that the substance generated through the reaction of nitrite ions with ammonium substances can adsorb into the cavities of cyclodextrin modified with thiol groups. This enhances the SERS signal intensity of nitrite derivatives and indirectly measures the concentration of nitrate ions. Under acidic conditions, the reaction between OPD and nitrite leads to the formation of a compound known as BTAH [64,65,66].

**Figure 1:**
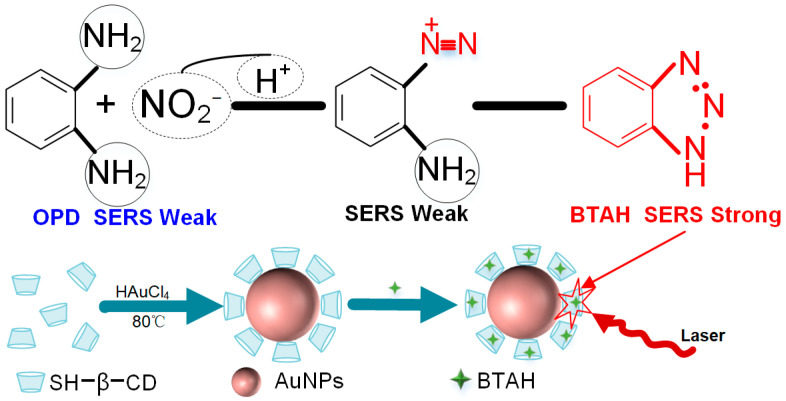
Schematic illustration of reaction of nitrite and OPD under acid conditions.

The reaction mechanism is illustrated in Figure 1. In this reaction, the SERS signals for BTAH, derived from nitrate ions, and SERS signals based on AuNPs are relatively weak. This is because BTAH cannot be directly adsorbed onto the surface of AuNPs. The literature reports suggest that large cyclic supramolecular compounds, such as cyclodextrin, can modify the surface of a nanomaterial substrate through covalent or non-covalent interactions, enhancing the functionality of the nanomaterial substrate [67,68]. In this experiment, thiol-modified cyclodextrin was employed as a stable ligand to modify the surface of AuNPs, resulting in the fabrication of a SH-β-CD@AuNPs functionalized nanomaterial substrate. The thiol groups of mono-(6-mercapto)-β-cyclodextrin used in this study readily bind to AuNPs. The modification with cyclodextrin significantly improves the ability of AuNPs to selectively recognize and adsorb target analytes. In aquatic environments, β-cyclodextrin serves as a large cyclic molecular receptor, allowing for certain organic and inorganic compounds and biomolecules to enter its cavity and form host–guest complexes. The derivative of nitrite ions, BTAH, demonstrates a strong affinity for the hydrophobic cavity of β-cyclodextrin, enabling selective entry [69,70]. Therefore, BTAH exhibits strong adsorption on the SH-β-CD@AuNPs substrate. Accordingly, in this study, gold nanoparticles (AuNPs) with a diameter of 50 nm and modified with cyclodextrin were synthesized for the detection of nitrogen derivatives, specifically BTAH, resulting from the conversion of nitrate ions to nitrites. Following the transformation of nitrate ions into nitrites and their subsequent reaction with OPD, BTAH exhibited a particularly distinct SERS signal on the surface of the SH-β-CD@AuNPs substrate.

### 3.2. Characterisation of SH-β-CD@AuNP Substrates

#### 3.2.1. UV–Vis Absorption Spectra

**Figure 2:**
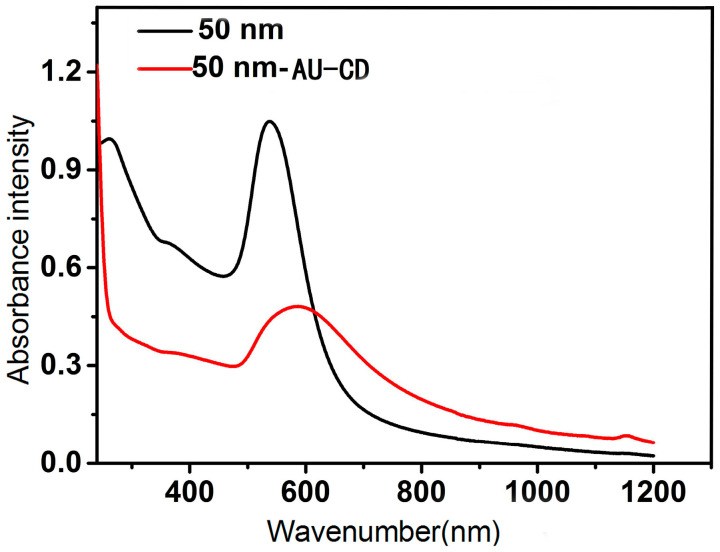
UV–vis spectrum absorption spectra of AuNPs and SH-β-CD@AuNPs.

A UV–visible absorption spectral characterization was carried out to gain a preliminary understanding of the AuNPs and SH-β-CD@AuNPs substrates (see Figure 2). It can be observed that the characteristic absorption peak of the AuNPs is approximately 520 nm, while the absorption peak of the synthesized SH-β-CD@AuNPs substrate is situated around 530 nm for the surface plasmonic resonance (SPR) absorption peak. The UV absorption peak position of AuNPs theoretically depends on the size and shape of the particles. Generally, gold nanoparticles exhibit a characteristic SPR absorption peak in the UV region. For spherical gold nanoparticles (50 nm), the surface plasmon resonance peak typically appears in the range of 520 to 530 nm. Smaller particles usually exhibit a blue shift, while larger particles may result in a red shift. The position in this range can serve as a preliminary indicator of the size of gold nanoparticles. 

Additionally, by comparing AuNPs and SH-β-CD@AuNPs, we observed that the peak disappeared after modifying with cyclodextrin, and the absorption peak position shifted to around 530 nm. This is because, in the absence of a modification with single-(6-thiol)-β-cyclodextrin, the AuNPs are stable due to electrostatic repulsion between the gold particles. After modification, the thiol groups on single-(6-thiol)-β-cyclodextrin form stable Au-S bonds, adsorbed on the surface of AuNPs [71]. This disrupts the original stable double electron layer structure, affecting the aggregation state of the gold sol. Therefore, after cyclodextrin modification, the UV–visible absorption spectrum of SH-β-CD@AuNPs shows a redshift of the absorption peak to 530 nm.

#### 3.2.2. Zeta Potential

**Figure 3:**
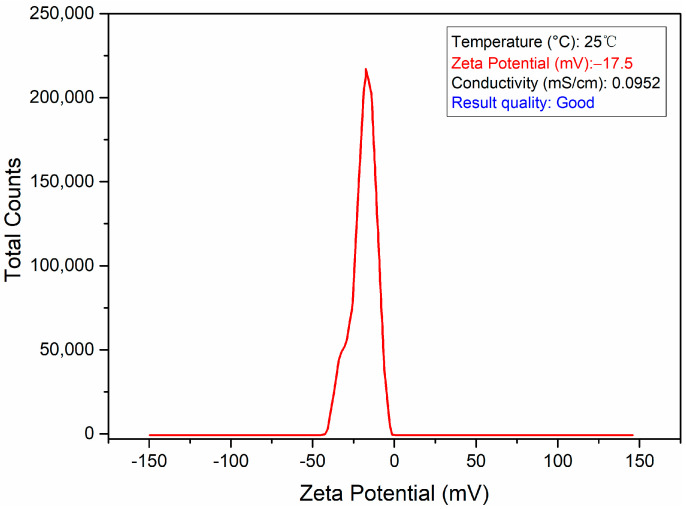
The zeta potential of SH-β-CD@AuNPs.

The zeta potential plot is the difference between the concentration and potential of the substance to be measured as a function of the distance from the charged surface of the particles suspended in the dispersed medium. The magnitude of the zeta potential represents the degree of electrostatic repulsion between neighboring charged particles in the dispersion [72,73]. Therefore, when the zeta potential (negative or positive) is high, the colloid stability is better, and when it is low, the colloid is easy to be deposited or flocculated. The zeta potential of the SH-β-CD@AuNPs nanomaterial substrate prepared in this paper is shown in Figure 3. The results show that the zeta potential is −17.5, which has good stability and better results in detection.

#### 3.2.3. TEM Characterization

**Figure 4:**
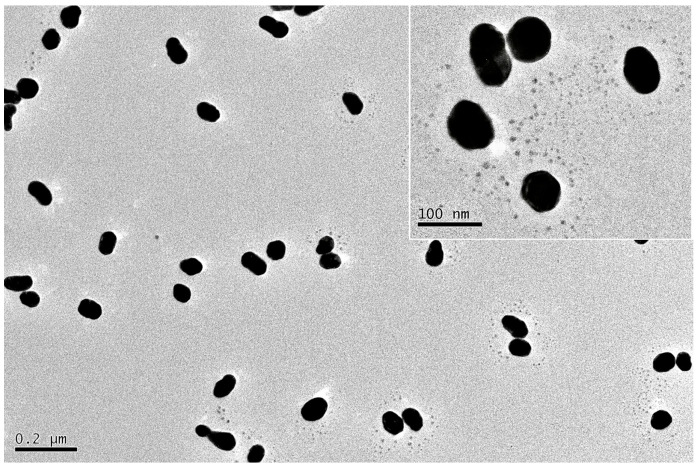
TEM images of SH-β-CD@AuNPs.

The external appearance, organization, and size distribution of the synthesized SH-β-CD@AuNPs substrates were characterized using transmission electron microscopy (TEM), and the results are presented in Figure 4. It reveals that the AuNPs exhibit relative homogeneity in size and dispersion, primarily adopting a spherical morphology. It is evident that there are black dot-like structures around the gold nanoparticles. This is attributed to the stable Au-S bond formed between the thiol group on single-(6-mercapto)-β-cyclodextrin (SH-β-CD) and the gold nanoparticles, disrupting the original stable double electron layer structure and influencing the aggregation state of the AuNPs. The reduced interparticle distance leads to aggregation. Furthermore, the UV–Vis absorption spectra before and after the modification with SH-β-CD also support the observation of aggregation. The original unmodified gold nanoparticles exhibit a characteristic resonance absorption peak at 520 nm, while the SH-β-CD-modified particles show a redshift in the absorption peak, indicating some degree of aggregation. Therefore, cyclodextrin has been successfully modified onto the gold nanoparticles.

**Figure 5:**
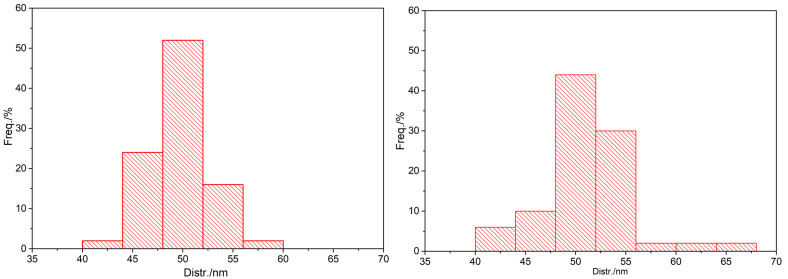
Size-distribution histogram of AuNPs (**left**) and SH-β-CD@AuNPs (**right**).

Additionally, using Nano Measure software1.2, we calculated the average particle sizes of AuNPs and SH-β-CD@AuNPs to be 49.3 nm and 50.1 nm, respectively. Figure 5 illustrates the size distribution of the AuNPs and SH-β-CD@AuNPs substrates obtained in this study. It is evident that more than 90% of the gold nanoparticles have sizes concentrated in the range of 45–55 nm, and for the SH-β-CD@AuNPs substrate, more than 70% have sizes within 50 ± 5 nm, with around 40% having sizes around 50 nm. In conclusion, the SH-β-CD@AuNPs substrate prepared in this study exhibits uniform particle sizes and excellent stability. The TEM characterization indicates that the modified nanomaterial substrates have been successfully prepared and exhibit good performance.

### 3.3. SERS Detection of Nitrate Nitrogen

#### 3.3.1. SERS Spectral Analysis

**Figure 6:**
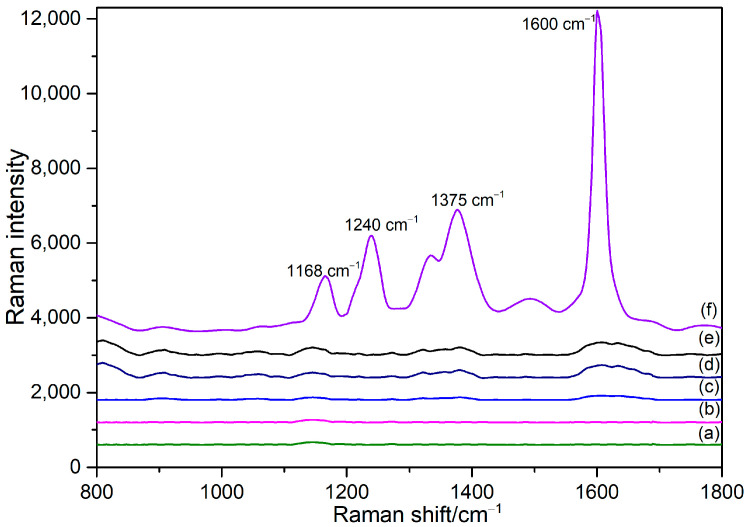
Characterization of the detection process. (a) SERS of OPD (100 μmol/L); (b) SERS of NaNO_3_ solutions (10 μmol/L); (c) SERS of NaNO_2_ solutions (10 μmol/L); (d) NRS of BTAH derived from the reaction between NO_2_^−^ and OPD; (e) SERS of BTAH on AuNPs; (f) SERS of BTAH based on SH-β-CD@AuNPs.

The SERS spectra of OPD solution with a concentration of 100 μmol/L, 10 μmol/L sodium nitrate solution, and 10 μmol/L sodium nitrite are shown in Figure 6 (a–c), respectively. It can be observed from the graphs that none of the three sample groups exhibit particularly obvious Raman characteristic peaks. These results indicated that none of the reactants involved in the cyclization reaction interfere with the SERS signals. In Figure 6 (d,e), it is evident that the Normal Raman spectra (NRS) and SERS on AuNPs of the nitrite ion and the OPD-derived substance BTAH also lack particularly distinct characteristic peaks.

Upon comparing Figure 6 (e,f), more noticeable Raman characteristic peaks appeared in the SERS of BTAH after mixing with SH-β-CD@AuNPs. In the absence of cyclodextrin, we observed the Raman spectra of BTAH and gold nanoparticles, finding that its Raman signals were weak. However, in the presence of β-cyclodextrin, the SERS signals of BTAH were significantly enhanced, with four distinct characteristic peaks at 1168, 1240, 1375, and 1600 cm^−1^. This result indicates that the introduction of cyclodextrin causes BTAH to enter the cyclodextrin cavity, forming a stable complex. The Raman characteristic peak at 1168 cm^−1^ is associated with the vibrational mode of the triazole ring, attributed to the triazole ring-breathing mode [74,75]. The peak at 1245 cm^−1^ corresponds to the planar bending vibration of C-H, and those at 1375 cm^−1^ represent the stretching vibration of the benzene ring and azole ring [75,76,77]. Additionally, a distinct characteristic peak at 1600 cm^−1^ is observed in Figure 6 (e), originating from the phenyl ring stretch vibration due to the inclusion complex formed by AuNPs with β-CD derivatives [78]. Furthermore, several weak characteristic peaks are visible in Figure 6, mainly encompassing the in-plane bending vibration of N-H and the stretching vibration of N=N=N.

The experimental results indicate that an interaction occurred between BTAH and the substrate of SH-β-CD@AuNPs, with BTAH entering the cavity of cyclodextrin in the SH-β-CD@AuNPs to form a new host–guest inclusion complex. These characteristic peaks indicate that the SH-β-CD@AuNPs synthesized in this paper greatly enhance the SERS signals of BTAH on its surface. Consequently, it can be used for the ultrasensitive detection of BTAH in mixed solutions, facilitating the simultaneous measurement of nitrate ion and nitrite ion concentrations.

#### 3.3.2. Calculation of Enhancement Factors

The enhancement of Raman spectral signal results from the increase of the effective cross-sectional area of the molecules, and the enhancement factor (EF) of SERS is correlated with the SERS cross-section [79]. The calculation of SERS-EF relies on the specific conditions of the SERS substrate, the target samples, and the excitation wavelength. The SH-β-CD@AuNPs nanomaterial substrate developed in this paper exists in colloidal form. Therefore, in this work, the chosen AEF calculation formula [80,81,82,83] is as follows:

(2) $$EF=\frac{I_{SERS}\times C_{RS}}{I_{RS}\times C_{SERS}}$$

where $I_{SERS}$ refers to the SERS spectrum of the BTAH molecule, the reaction product of nitrite conversion of nitrate to OPD phantom. $C_{RS}$ refers to the concentration of nitrite nitrogen (mol/L) at the time of detection in the NRS of BTAH, while $I_{RS}$ refers to the NRS of BTAH. $C_{SERS}$ refers to the concentration of nitrite nitrogen (mol/L) as detected by the SERS of BTAH.

The SERS-EF values at SERS characteristic peaks at 1168, 1240, 1375, and 1600 cm^−1^ were calculated by substituting the experimentally measured values of Raman spectral intensity and concentration into the aforementioned formula. As illustrated in Table 1, the calculated results demonstrate that the SH-β-CD@AuNPs nanomaterial substrate exhibits a robust SERS signal with a significant enhancement effect when detecting nitrite ion derivatives. Consequently, the modified nanomaterial substrate developed in this study will display heightened sensitivity in a practical nitrate measurement.

#### 3.3.3. Stability and Reproducibility of the SH-β-CD@AuNPs Substrate

**Figure 7:**
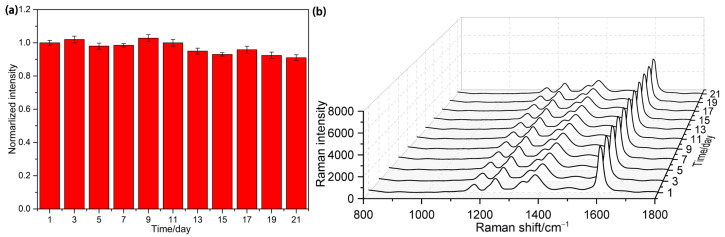
(**a**) Changes in SERS intensity of nitrate ion derivatives at 1660 cm^−1^; (**b**) SERS spectra of nitrate ion derivatives at the same concentration within three weeks.

Currently, nanomaterials have emerged as a key focus in the realm of high-sensitivity and high-reliability electrochemical sensors, optical sensors, and biosensors. Nevertheless, challenges such as nanomaterial agglomeration, stability, and reproducibility during preparation persist. Metal nanomaterials particularly exhibit high reactivity and are susceptible to oxidation during use, resulting in decreased SERS substrate activity. Additionally, the stability of various nanomaterials substrate can vary widely. Consequently, this paper evaluates the stability of the SH-β-CD@AuNPs substrate. An amount of 10 μmol/L nitrate ion was selected for this assessment. The prepared SH-β-CD@AuNsP substrate was employed to monitor the nitrate ion samples with the same concentration over three consecutive weeks, as depicted in Figure 7a. It can be observed that the SERS spectrum of 10 μmol/L nitrate ion, as measured by the present method, exhibited no significant changes over the three-week period. Figure 7b illustrates the variation in the intensity of the selected SERS characteristic peak located at 1600 cm^−1^ over 21 days. Notably, the intensity of the SERS characteristic peak at 1600 cm^−1^ showed minimal change over 21 days, with a relative standard deviation of 9.63%. These experimental results affirm the good stability of the SH-β-CD@AuNP substrates prepared in this study.

**Figure 8:**
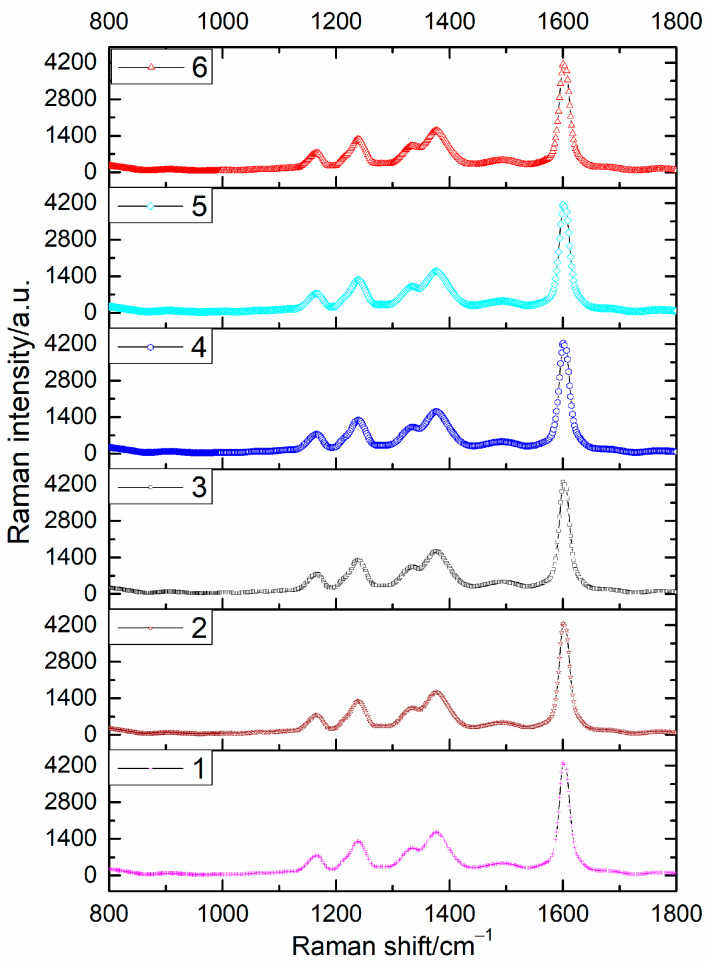
Repeatability of SH-β-CD@AuNP substrate prepared in different batches.

Moreover, the reproducibility of different batches of the SH-β-CD@AuNP substrates for nitrate detection was explored, and the results are presented in Figure 8. Notably, the SERS of BTAH on SH-β-CD@AuNP substrates prepared in six batches did not show significant changes in the detection of the same concentration of nitrate ions. These findings prove the excellent reproducibility of the preparation method in this study.

### 3.4. Optimization of Experimental Conditions

#### 3.4.1. Effect of pH

**Figure 9:**
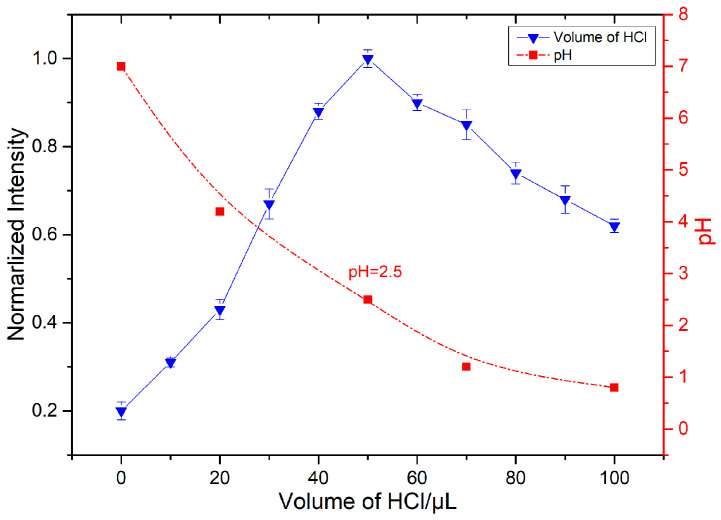
The influence of pH.

It has been reported in the literature that the cyclization reaction of nitrite with OPD to produce BTAH exclusively takes place under acidic conditions. Under alkaline and neutral conditions, the reaction of nitrite with OPD does not proceed. Therefore, the pH level plays a crucial role in determining the rate of the cyclization reaction and whether nitrite is fully derivatized to BTAH. In this study, HCl and NaOH were employed to adjust the pH of the reaction system. Under the same experimental conditions, a sodium nitrate solution with a concentration of 10^−6^ mol/L was chosen for conversion to sodium nitrite. Subsequently, varying volumes of a HCl working solution were added. The SERS of the mixed solutions at different pH values were obtained using the above method, and the changes in the intensity of the SERS characteristic peaks at 1660 cm^−1^ were compared across different sample groups. As shown in the Figure 9, when adding 0–100 μL HCl, the SERS signal of the reaction product first increases and then decreases. The maximum intensity of the 1600 cm^−1^ SERS characteristic peak is observed when adding 50 μL of HCl, corresponding to a pH of approximately 2.5. In summary, the reaction reached its optimal conditions at pH 2.5. Therefore, 50 μL of the HCl working solution were introduced into the solution system to ensure the smooth progression of the cyclization reaction and the accuracy of the detection results.

#### 3.4.2. Effect of Reaction Temperature and Time

**Figure 10:**
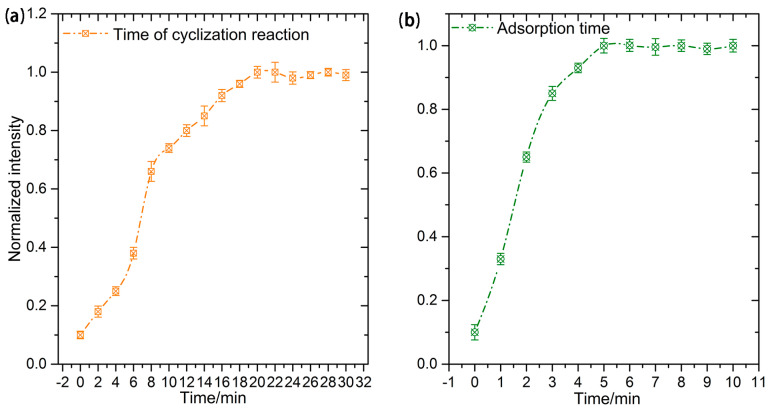
(**a**) The influence of cyclization reaction time; (**b**) The influence of adsorption time of BTAH on SH-β-CD@AuNPs substrate.

In this paper, the effects of temperature and reaction time on nitrate conversion experiments, the cyclization reaction of nitrite, and the adsorption properties of BAH on SH-β-CD@AuNPs substrates are investigated. The previous literature indicates that temperature plays a crucial role in the rate of nitrate conversion. Under room temperature conditions, the complete conversion of nitrate to nitrite using VCl_3_ typically takes 10 h. We chose a temperature of 50 °C and employed sealed heating in a drying oven for 30 min to ensure the thorough conversion of nitrate to nitrite in our experiments [84]. Regarding the cyclization reaction of OPD with nitrite, the temperature requirements are not particularly stringent. Here, we selected a room temperature of 23 °C for the nitrite cyclization reaction. Exploring the impact of the cyclization reaction time, we investigated the effects within the 0–30 min time range. As shown in Figure 10a, the SERS intensity of the reaction product BTAH exhibited significant changes at the initial stages of the reaction, gradually enhancing. Around the 20 min mark, the SERS intensity began to stabilize, reaching its peak at 20 min, with no further significant alterations. Therefore, the cyclization reaction time between OPD and nitrite was set at 20 min.

Additionally, the effects of temperature and time on the adsorption of BTAH with the substrate of SH-β-CD@AuNPs were also explored. The substrate of SH-β-CD@AuNPs forms a host–guest inclusion complex with the guest molecule at room temperature. At higher temperatures, BTAH is detached from the cavities of cyclodextrins in SH-β-CD@AuNPs. Within the normal temperature range, there is little effect on the host–guest inclusion complex. Hence, this study designates room temperature as the optimal condition for the adsorption of BTAH onto the SH-β-CD@AuNPs substrate. Regarding the effect of adsorption time, the SERS intensity of the reaction products within the range of 0–10 min was explored. As shown in Figure 10b, within the first 5 min of mixing BTAH with the SH-β-CD@AuNPs substrate, the SERS intensity of the reaction product notably increased. The intensity peaked at 5 min and remained unchanged thereafter. This observation confirms that, at the 5 min mark, all the generated BTAH in the mixing solution had entered the cavities of cyclodextrins on the SH-β-CD@AuNPs substrate. Consequently, the optimum reaction time for the interaction between BTAH and the SH-β-CD@AuNPs substrate was determined to be 5 min in this study.

In summary, the optimal temperature for the nitrate conversion, the cyclization reaction of nitrite, and the adsorption of BTAH onto the SH-β-CD@AuNPs substrate were 50, 23, and 23 °C, respectively. The optimal reaction times were 30, 20, and 5 min, respectively.

#### 3.4.3. Effect of OPD Concentration

**Figure 11:**
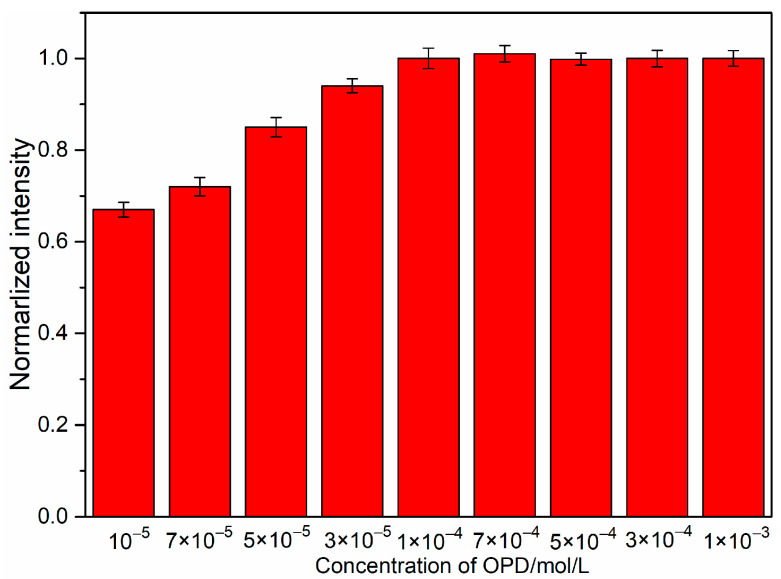
The influence of different concentrations of OPD solutions on the SERS intensity at 1600 cm^−1^.

In the cyclization reaction between OPD and nitrite ions, the concentration of OPD directly affects whether the reaction can proceed to completion, thus impacting the detection results of nitrite nitrogen. We chose a nitrite ion concentration of 10^−5^ mol/L to examine the effect of different concentrations of the OPD solution on the measurement results. As illustrated in Figure 11, the change in the intensity of the SERS characteristic peak at 1600 cm^−1^ for the generated BTAH was observed when adding 200 μL of the OPD solution with concentrations ranging from 10^−3^ to 10^−5^ mol/L. It can be seen from the figure that the intensity of the SERS peaks at 1600 cm gradually increased as the concentration of the OPD solution rose from 10^−5^ mol/L to 10^−4^ mol/L. The intensity of the SERS peaks at 1600 cm^−1^ reached its maximum value at an OPD concentration of 10^−4^ mol/L, and the SERS peaks remained constant when the concentration of the OPD solution was in the range of 10^−4^ to 10^−3^ mol/L. Beyond this range, the intensity of SERS no longer changed for OPD concentrations ranging from 10^−3^ to 10^−1^ mol/L. Therefore, an OPD solution concentration of 10^−4^ mol/L was selected as the optimal concentration for the SERS detection of nitrate ions.

#### 3.4.4. Effect of Substrate Concentration

**Figure 12:**
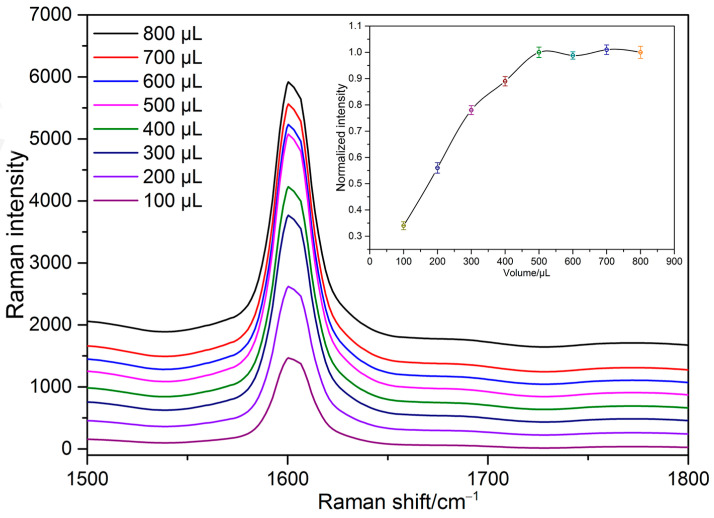
SERS spectra of the mixed solution with different concentrations of SH-β-CD@AuNPs.

We also investigated the impact of varying amounts of SH-β-CD@AuNPs substrates on the adsorption performance of BTAH. According to our experiment’s results, the concentration of SH-β-CD@AuNPs is 50 μg/mL. Under the same experimental conditions, 200 μL of sodium nitrate solution with a concentration of 10^−5^ mol/L was converted into a sodium nitrite solution following the procedure outlined in this paper. Subsequently, 200 μL of the OPD solution was added to undergo the cyclization reaction, and different volumes of the SH-β-CD@AuNPs substrate solution were introduced to compare their effects on the experimental outcomes. From Figure 12, it can be depicted that the intensity of the SERS characteristic peak at 1600 cm^−1^ showed an obvious increasing trend within the SH-β-CD@AuNPs substrate concentration range of 2.5–20 μg/mL. The intensity of the SERS characteristic peak at 1600 cm^−1^ reached its maximum at 12.5 μg/mL. Subsequently, the SERS intensity reached equilibrium and ceased to change within the range of 12.5–20 μg/mL. Therefore, 12.5 μg/mL (500 μL) SH-β-CD@AuNPs were chosen as the optimal concentration of SH-β-CD@AuNPs for nitrate ion detection in this paper.

### 3.5. Analytical Performance

#### 3.5.1. Selectivity

**Figure 13:**
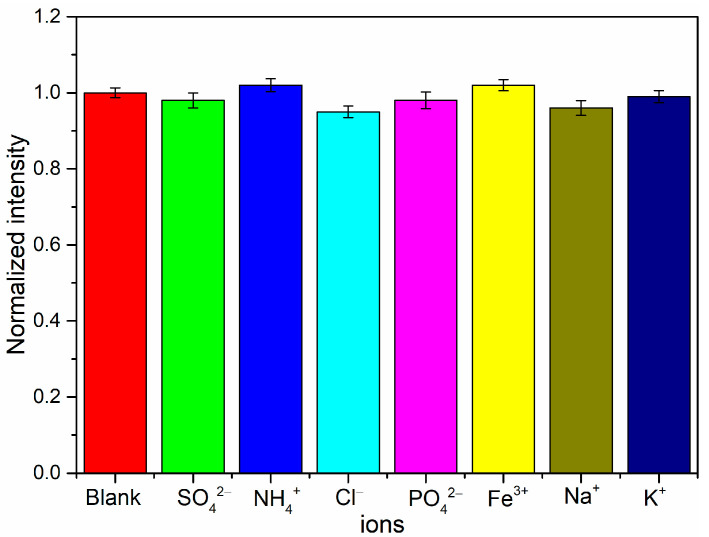
The influence of interfering ions.

The aquaculture water is inherently dynamic, encompassing ions such as CI^−^, SO_4_^2−^, NH_4_^+^, PO_3_^−^, Fe^3+^, Mg^2+^, and coexisting organic matter, along with impurities. Therefore, the effects of common interfering ions in aquaculture water on the measurement results of nitrate ions were discussed. The interference from nitrite ions is discussed below. A nitrate ion concentration of 10^−5^ mol/L was selected for the interference ion experiment in this paper. The blank group test samples and samples with the addition of specific concentrations of interference ions (CI^−^, SO_4_^2−^, NH_4_^+^, PO_4_^2−^, Fe^3+^, Mg^2+^, Na^+^, and K^+^) were measured under the same experimental conditions. Subsequently, the intensity of a SERS characteristic peak at 1600 cm^−1^ was measured for each group of mixed samples, and the experimental results are presented in Figure 13. From the figure, it can be observed that the concentration of the added interfering ions is 100 times that of nitrate ions. In the paper, whether it is sodium nitrate, potassium nitrate, or nitrate X, the actual species participating in the reaction is the nitrate ion. These cations have no impact on the reaction between OPD and nitrite ions. Additionally, these ions cannot be adsorbed onto gold nanoparticles or substituted for BTAH within the cyclodextrin cavity. Therefore, their presence has minimal influence on the SERS signal of BTAH. The relative standard error is less than 5.67%. This suggests that, based on the cyclization reaction between OPA and nitrite ion, the prepared SH-β-CD@AuNPs substrate has high selectivity for detecting nitrate ions in complex water environments.

#### 3.5.2. Linearity and LOD

**Figure 14:**
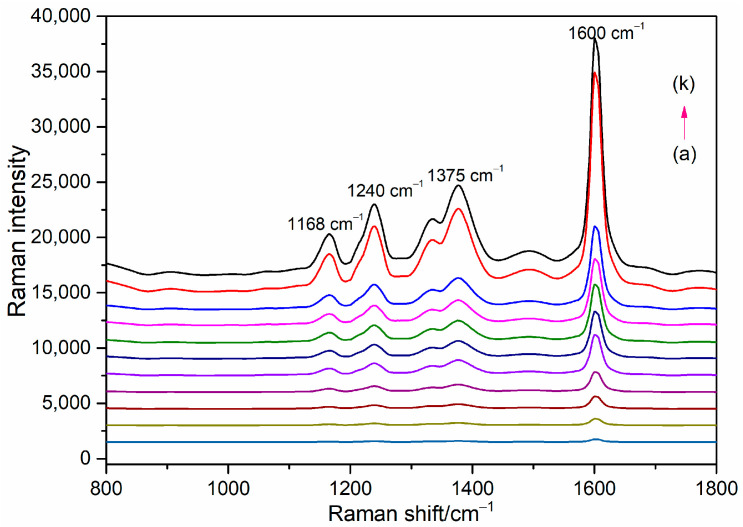
SERS spectra of BTAH at different concentrations of nitrate ions: (a) 0.1, (b) 0.3, (c) 0.5, (d) 0.7, (e) 1, (f) 3, (g) 5, (h) 7, (i) 10, (j) 30, (k) 50 μmol/L.

**Figure 15:**
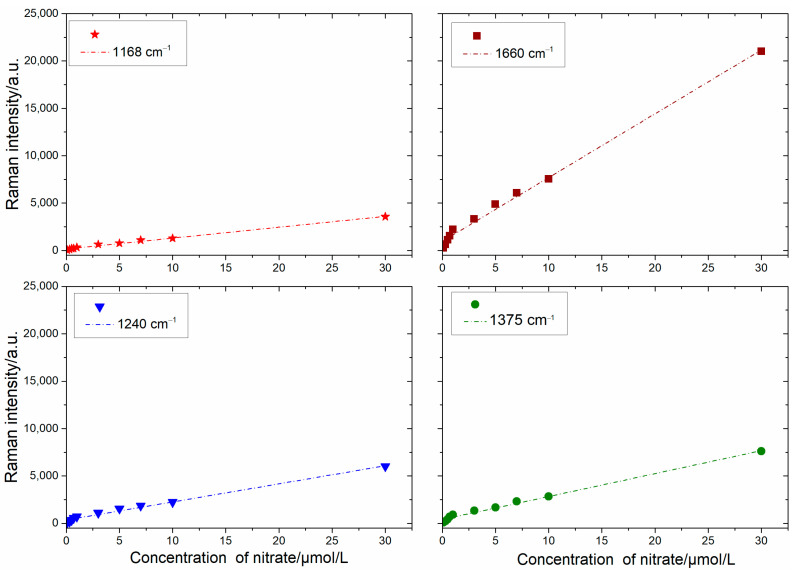
The calibration curves at 1168, 1240, 1375, and 1600 cm^−1^.

Under the optimal experimental conditions, 11 groups of nitrate ion samples in the range of 0.1–50 μmol/L were detected. It can be seen from Figure 14 that the SERS spectra obtained from different concentrations of nitrate ions exhibited prominent Raman characteristic peaks at 1168, 1240, 1375, and 1600 cm^−1^. Even at the lowest nitrate ion concentration of 0.1 μmol/L, BTAH displayed a weak SERS signal. Therefore, the Raman characteristic peaks at 1168, 1240, 1375, and 1600 cm^−1^ were selected for nitrate ion detection. The linear regression models were established for SERS intensity and nitrate ion concentration. According to the least squares method, the calibration curves obtained based on the relationship between the intensities of four SERS characteristic peaks and the concentration of nitrate are illustrated in Figure 15. Remarkably, these curves demonstrate a good linear relationship within the concentration range of 0.1–30 μmol/L. The calibration curve of the four SERS characteristic peaks was calculated by the least squares method, and the linear regression equations and correlation coefficients were summarized in Table 2. Therefore, the nitrate concentration could be accurately determined using the regression equations. The experimental results showed that the proposed SH-β-CD@AuNPs substrate exhibits a highly reliable linear relationship for nitrate ion concentrations in the range of 0.1–30 μmol/L.

To investigate the lowest measured concentration, 20 groups of blank concentration samples were detected, and the lowest detection limits (LOD) at the four characteristic peaks of 1168, 1240, 1375, and 1600 cm^−1^ were calculated at a signal-to-noise ratio of S/N = 3 [85]. The results were 3.33 × 10^−2^, 5.84 × 10^−2^, 2.40 × 10^−2^, and 1.05 × 10^−2^ μmol/L, respectively, as shown in Table 2. Now, a few more methods based on optical and electrochemical for nitrate determination developed in recent years, and the detection parameters, such as the materials, principle, LOD, and linear range, have been added in Table 3. The proposed SERS detection method of nitrate on SH-β-CD@AuNPs has higher sensitivity, higher selectivity, and a lower LOD. Therefore, the development of highly sensitive and selective SERS substrates is crucial for achieving the rapid and accurate detection of low concentration nitrate nitrogen in aquaculture.

#### 3.5.3. Detection of Nitrate Nitrogen in Real Water Environment

To verify the reliability of this nitrate nitrogen detection method in an actual aquaculture water body, four actual water environment samples were selected for testing. These samples encompassed the water bodies of a grouper marine aquaculture workshop, a fish tank for carp aquaculture, pure water, and seawater. The testing protocol entailed two sets of experiments for each sample: one for nitrite nitrogen detection in the actual samples and the other for total nitrite nitrogen after a nitrate nitrogen reduction. Each group of samples underwent five tests, and the average value derived from these experiments was taken as the final result. The SERS spectra data were obtained by subjecting the mixed solutions to a SERS analysis. Specifically, the SERS signal intensity at 1600 cm^−1^ was selected and substituted into the univariate linear regression equation in Table 2.

Furthermore, a spiked recovery experiment was conducted to evaluate the accuracy of the method under optimal experimental conditions. As depicted in Table 4, the results demonstrate that this method aligns with the requisites for nitrate ion detection, exhibiting spiked recoveries ranging from 96.8% to 103.4% and relative standard deviations between 1.89% and 4.21%. These findings underscore the method’s robust detection accuracy and its applicability for detecting nitrate ion concentrations in actual aquaculture water, facilitating the simultaneous detection of nitrate nitrogen and nitrite nitrogen.

## 4. Conclusions

In this paper, a nanomaterial substrate of SH-β-CD@AuNPs was synthesized, which exhibits a rapid, highly sensitive, and selective SERS approach for the detection of nitrate nitrogen in water. This method leverages the cyclization reaction between nitrite and OPD on the SH-β-CD@AuNPs substrate. Several experimental parameters, including the pH, temperature, reaction time, reactant concentration, and concentration of SERS substrate, were optimized to achieve optimal conditions. Under the optimal conditions, the nitrate derivative BTAH exhibited distinct SERS characteristic peaks at 1168, 1240, 1375, and 1600 cm^−1^, with LOD of 3.33 × 10^−2^, 5.84 × 10^−2^, 2.40 × 10^−2^, and 1.05 × 10^−2^ μmol/L, respectively. The linear range for nitrate ion detection spanned 0.1–30.0 μmol/L. Additionally, the method demonstrated accuracy and spiked recoveries ranging from 96.8% to 103.4% in the analysis of real samples.

The experimental results indicate that the proposed method has high sensitivity and selectivity, meeting the requirements for the accurate detection of nitrate nitrogen and nitrite nitrogen in aquaculture water. Future research will focus on the “dual-mode” detection of nitrate ions, incorporating both SERS and fluorescence based on the SH-β-CD@AuNPs substrate. This approach aims to enhance the detection accuracy, sensitivity, and selectivity by utilizing the improved substrate as both SERS and fluorescence probes.

## Figures and Tables

**Table 1:** The calculated results of SERS-EF.

Raman Characteristic Peaks (cm^−1^)	SERS-EF
1168	2.11 × 10^6^
1240	4.67 × 10^6^
1375	1.78 × 10^7^
1600	1.98 × 10^8^

**Table 2:** The linear regression results and LOD.

Raman Characteristic Peaks (cm^−1^)	Linear Regression Equations	R^2^	LOD (μmol/L)
1168	*y* = 114.78*x* + 265.81	0.9924	3.33 × 10^−2^
1240	*y* = 190.9*x* + 363.61	0.9890	5.84 × 10^−2^
1375	*y* = 241.96*x* + 423.95	0.9918	2.40 × 10^−2^
1600	*y* = 672.67*x* + 979.02	0.9945	1.05 × 10^−2^

**Table 3:** Comparison of the performance from different detection methods.

Method	Materials	Linear Range (μM)	LOD (μM)	Ref.
Voltammetric	ERGO-MWCNT a	10–750	3.3	[8]
Potentiometric	Ni_2_O_3_/rGO	10–2400	27.4	[13]
Voltammetric	CdS nanorods on glass screen print electrode	50–5000	2.3	[14]
Ion Chromatography	N/A	0.1–3	3 × 10^−2^	[20]
SERS	Halloysite nanotubes-AgNPs	10^2^–10^5^	2 × 10^−2^	[86]
SERS	SH-β-CD@AuNPs	0.1–30	1.05 × 10^−2^	This work

a ERGO-MWCNT: graphene oxide-multiwalled carbon nanotubes.

**Table 4:** Determination of nitrate in different water samples by the present method and spectrophotometry.

Sample	Added (μmol/L)	Spectrophotometry (μmol/L)	This Work (μmol/L)	RSD (%)	Recovery (%)
Carp fish tank	0	8.12	5.17	2.14	103.4
	5.0	9.90	10.34	3.75	
Land-based factories (grouper)	0	25.09	24.10	3.24	96.8
	5.0	29.87	28.94	2.70	
Sea	0	10.98	10.65	2.57	102.8
	5.0	11.63	15.79	3.41	
River	0	36.2	31.09	N/A	N/A
	5.0	41.6	31.4	N/A	

## Data Availability

All data are available from the corresponding author upon reasonable request.
